# HIV-1 latent infection modulates RNA sensors and innate immunity signaling in human brain pericytes

**DOI:** 10.21203/rs.3.rs-7087301/v1

**Published:** 2025-09-22

**Authors:** Daniel Adesse, Gabriella Mezzich, Prem Parikh, Roberto Labrada, Paloma Carvalho Vieira, Anne Caroline Marcos, Silvia Torices, Michal Toborek

**Affiliations:** Instituto Oswaldo Cruz; University of Miami Miller School of Medicine; University of Miami Miller School of Medicine; University of Miami Miller School of Medicine; Instituto Oswaldo Cruz; Instituto Oswaldo Cruz; University of Miami Miller School of Medicine; University of Miami Miller School of Medicine

**Keywords:** HIV-1 latency, Brain pericytes, RNA sensors, RIG-I, MDA5, cGAS-STING pathway, Innate immunity, Type I interferons, Blood-brain barrier, Neuroinflammation

## Abstract

**BACKGROUND.:**

Cerebrovascular and neurological diseases are frequent co-morbidities among people with HIV. Our laboratory has described that brain pericytes (BP) can harbor HIV infection and function as a reservoir of viral latent infection in the Central Nervous System. Given that the interaction between pericytes and endothelial cells in the blood-brain barrier (BBB) are necessary for proper formation, development, stabilization, and maintenance of the BBB, we investigated whether active and/or latent HIV-1 infection can differently modulate inflammatory response in BPs, with a focus on RNA sensor and type I interferon (IFN) pathways.

**METHODS.:**

Primary cultures of human brain vascular pericytes were infected with 60 ng/ml of p24 HIV (NL4–3 strain). Three and 7 days post-infection (dpi), which were previously shown to correspond to active and latent infection, respectively, protein and total RNA were extracted from infected and uninfected control cultures. Expression or protein content of RNA sensors and downstream activators and members of type I IFN response and IFN response genes were determined by RT-qPCR and western blotting, respectively.

**RESULTS.:**

At 3 dpi, HIV gag transcripts were detected at high levels in infected cultures, which dropped significantly at 7 dpi. RIG-1 protein levels were significantly increased at 7 dpi (2.3-fold), whereas MDA5 was decreased by 0.5-fold. Similarly, STING (Stimulator of IFN genes) mRNA and protein levels were increased at 7 dpi, accompanied by increased expression of MAVS and of TBK1 (TANK-binding kinase 1, 1.7-fold), and phosphorylation of IRF3 (interferon regulatory factor 3). The IFN-α/β receptor type I (IFNAR1) and STAT1 transcripts were selectively increased at 7 dpi. Expression of interferon-induced protein with tetratricopeptide repeats-1 (IFIT1) was transiently increased at 3 dpi and reduced at 7 dpi. No changes in the cGAS, TRAF3, IFN-stimulated gene 15 (ISG15) and Interferon-induced GTP-binding protein Mx1 were observed at the mRNA levels in infected cultures, as compared to controls.

**CONCLUSIONS.:**

HIV-1 differentially modulates IFN responses in BPs, which can affect BBB integrity in chronic infections. Interestingly, latently infected BPs can contribute to long-term neuroinflammatory stimuli in the CNS.

## INTRODUCTION

One of the major obstacles to achieving an HIV cure is the formation of viral reservoirs. HIV-1 can integrate into the host genome, allowing low-level replication in latent reservoirs, leading to potential rebounds of viremia and/or contributing to the development of HIV-related comorbidities. The main HIV reservoirs in the body are CD4+ T cells; however, there are other cell reservoirs, e.g., hematopoietic stem cells, dendritic cells, and the cells of the CNS that are less understood and recognized (reviewed in [1]). Due to the high incidence of HIV-1-associated neurological disorders, defining the nature of CNS reservoirs is of critical relevance to translational medicine and clinical practice. The CNS reservoirs are particularly important because they are anatomically protected by the blood-brain barrier (BBB), which limits antiretroviral drug penetration and the efficient treatment of HIV-1 infection of the brain. Microglial cells make up the main reservoir of HIV-1 within the CNS [2]. However, other cell types, such as perivascular macrophages and astrocytes, have also been studied as potential hosts for latent HIV-1 reservoirs within the CNS.

Following infection by HIV-1, the virus quickly propagates and infects several tissues, including the brain. It has been demonstrated that HIV-1 reaches the CNS shortly after infection and can create CNS reservoirs [3–5]. Alterations of the blood-brain barrier (BBB) occur in the earliest stages of HIV-1 infection [3–14] and then persist throughout disease progression. Brain infection by HIV-1, along with BBB dysfunction, has been linked to the several co-morbidities observed in people with HIV (PWH), such as cerebrovascular disease and neurocognitive problems. Mounting evidence demonstrates that the CNS is an important HIV-1 reservoir that needs to be addressed for patient wellness, therapy, and cure.

The BBB is formed by brain microvessels and is positioned at the interface between the blood and the CNS. Functionally, the BBB is created by an interactome between endothelial cells, pericytes, perivascular astrocytes, microglia, and neurons, which is called the neurovascular units (NVU). Brain pericytes are multifunctional cells wrapped around the brain endothelium via cytoplasmic processes that extend along the abluminal surface of the endothelium. Part of the pericyte-endothelial interface is separated by the basement membrane [15], which is actively remodeled by both pericytes and endothelial cells during angiogenesis [16], development [15], and tumorigenesis [17]. However, pericytes remain also in direct contact with endothelial cells [18]. Recent evidence on pericyte ontogeny identified that a substantial subpopulation of brain pericytes originates from myeloid progenitors [19].

The majority of HIV-1 replication in the brain occurs in microglial cells and perivascular macrophages [2,20,21]. In addition, astrocytes can be productively infected and may play a role as an HIV reservoir [22]. Importantly, our group previously demonstrated that brain pericytes are not only susceptible to HIV-1 infection but also function as a latent reservoir within the CNS [23]. These findings were confirmed in subsequent studies by us [24–28] and, independently, by another group [29]. In addition, they contributed to vigorous research on the involvement of pericytes in HIV-1 infection [30–36]. Brain pericytes can also act as a reservoir for latent HIV-1 [26] and that latent infection alters pericyte biology, including gap junction [25] and tight junction [27,28] communication and signaling, and may lead to BBB disruption [23]. Moreover, active HIV-1 infection alters histone deacetylase Sirtuin-1 activity [26] and innate immune responses [37].

HIV-1 infection activates innate immune responses through RNA sensing pathways, leading to the production of type I interferons (IFN-I), particularly IFN-α and IFN-β, which play critical roles in antiviral defense [38]. Cellular pattern recognition receptors (PRRs) such as retinoic acid-inducible gene I (RIG-I) and melanoma differentiation-associated protein 5 (MDA5) detect HIV-1 single-stranded RNA (ssRNA) in the cytoplasm, triggering downstream signaling through mitochondrial antiviral-signaling protein (MAVS) and TANK-binding kinase 1 (TBK1), activated by stimulator of interferon genes (STING), to activate further interferon regulatory factors (IRF3 and IRF7), culminating in IFN-I production [39]. These pathways are also active in human brain pericytes [37]. Once secreted, IFN-α and IFN-β bind to their receptors (IFNAR1/IFNAR2), stimulating the JAK/STAT pathway, which upregulates a range of interferon-stimulated genes (ISGs) known to restrict viral replication [38]. However, HIV-1 has developed evasion mechanisms to suppress innate immune activation, including shielding its viral RNA within the capsid and disrupting IFN-I signaling through viral accessory proteins [39]. The ability of HIV-1 to persist in the CNS is particularly concerning, as brain pericytes serve as a latent reservoir, potentially maintaining a prolonged inflammatory state. While pericytes have been identified as an HIV-1 reservoir, the mechanisms governing their transition from active to latent infection remain poorly understood. Investigating how HIV-1 modulates innate immune responses in pericytes is critical for understanding their role in BBB dysfunction and neuroinflammation.

## METHODS

### 1. Cell culture and experimental design

Human brain pericytes (ScienCell, Carlsbad, CA, USA, Cat# 1200) were maintained with specific cell culture media and used for experiments between passages 6 and 8. For RNA or protein extraction assays, cells were plated onto 6-well plates at a density of 500.000 cells/well. 24 hours after plating, cells were infected with HIV-1 (NL-43 strain) at a concentration of 60 ng p24 HIV/ml in 1 ml of fresh pericyte media.

### 2. HIV-1 production and infections

HIV pNL4–3 plasmid obtained from the NIH AIDS Reagent Program (Division of AIDS, NIAID, National Institutes of Health) was employed. HIV pNL4–3 plasmid was amplified using Stbl3 competent cells (Thermo Fisher Scientific, Carlsbad, CA, USA, Cat# C737303) and isolated using PureYield Plasmid midi-prep system (Promega, Madison, WI, USA, Cat# A2492). Viral stocks were created as described [27]. Briefly, a total of 50 μg of proviral plasmid was transfected into 10^7^ human HEK293T/17 cells (ATCC, Manassas, VA, USA, Cat# CRL-11268) using Lipofectamine 2000 (Thermo Fisher Scientific, Cat# 11668–027). Media was changed to fresh Opti-Mem (Thermo Fisher Scientific, Cat# 11058–021) 18 hours post transfection and cells were incubated for additional 48 hours. Supernatant was then collected and filtered through 0.45 μm-pore size (Millipore Sigma, Massachusetts, MA, USA, Cat# 430314) filters to remove cell debris. Supernatants were concentrated using 50 kDa molecular weight exclusion columns (Millipore Sigma, Cat# UFC905024) and the aliquots were stored at −80°C.

### 3. RT-qPCR

Total RNA was extracted at desired time points with RNeasy micro kit (Qiagen, Hilden, Germany, EU, Cat# 74104), following manufacturer’s instructions. RT-qPCR were performed with 100 ng of RNA using the qScript XLT 1-Step RT-qPCR ToughMix Low ROX (Quantabio, Beverly, MA, USA, Cat #89236–676) reaction mix and the Applied Biosystems 7500 system (Applied Biosystems, Foster City, CA). TaqMan Gene Expression Assays and the following primers were used for gene amplification: DDx58 (RIG-1, Hs01061433_m1), MDA5 (IFIH1, Hs00223420_m1), MAVS (Hs00920075_m1), IRF3, IRF7 (Hs01287244_cn), TBK1 (Hs00975471_m1), STING (Hs00736955_g1), cGAS (Hs00403553_m1), TRAF3 (Hs00936781_m1), IFN α5 (Hs03044218_g1), IFN β (Hs01077958_s1), IFNAR1 (Hs01066116_m1), IFNAR2 (Hs01022059_m1), STAT1 (Hs01013996_m1), IFIT1 (Hs03027069_s1), ISG15 (Hs00192713_m1), MX1 (Hs00895608_m1). Quantitative analyses were performed using the 2^−ΔΔct^ method.

### 4. Western Blotting

Protein lysates were obtained by scraping the cell monolayers in the presence of 80 μL RIPA lysis buffer (Santa Cruz Biotechnology, Dallas, TX, USA, Cat# sc-24948a) with protease inhibitors, PMSF and sodium orthovanadate. Samples were incubated at 4 °C for 25 minutes, being homogenized with vortex every 5 minutes and centrifuged for 15 min at 4 °C at 14.000 rpm. Supernatants containing proteins were transferred to new tubes and protein concentration was determined using BCA protein kit (ThermoFisher, Cat# 23223). Fifteen μg of protein were loaded onto 4–20% Mini-PROTEAN TGX Stain-Free Protein Gels (Bio-Rad, Hercules, CA, USA,) and separated by electrophoresis at 100 V for 90 minutes. Then, proteins were transferred to Trans-Blot Turbo Midi 0.2 μm nitrocellulose transfer packs (Bio-Rad, Cat# 170–4159) and blocked for one hour with 5% bovine serum albumin in TBST (TBS with 0.01% Tween 20), followed by incubation overnight with primary antibodies at 4 °C. Membranes were then washed with TBST and incubated with secondary antibodies for 1 h at 20 °C and scanned with Licor CLX imaging system and the Image Studio 4.0 software (LI-COR). Anti-GAPDH antibody (1:20000, Novus Biologicals, Woburn, MA, USA, Cat# NB600–502FR or Cat# NB600–5021R) was used as loading control. The list of antibodies and dilutions used herein are in Table 1.

### 5. Statistical analyses

Statistical analyses were performed with GraphPad Prism Software v.10.3.1 (La Jolla, CA, USA). For gene or protein expression levels we used Two-Way ANOVA test with Fisher’s LSD test for multiple comparisons. p values <0.05 were considered as statistically significant.

## RESULTS

### 1. Active and latent HIV-1 infection differentially regulates the antiviral RIG-I pathway in human brain pericytes

**Figure 1:**
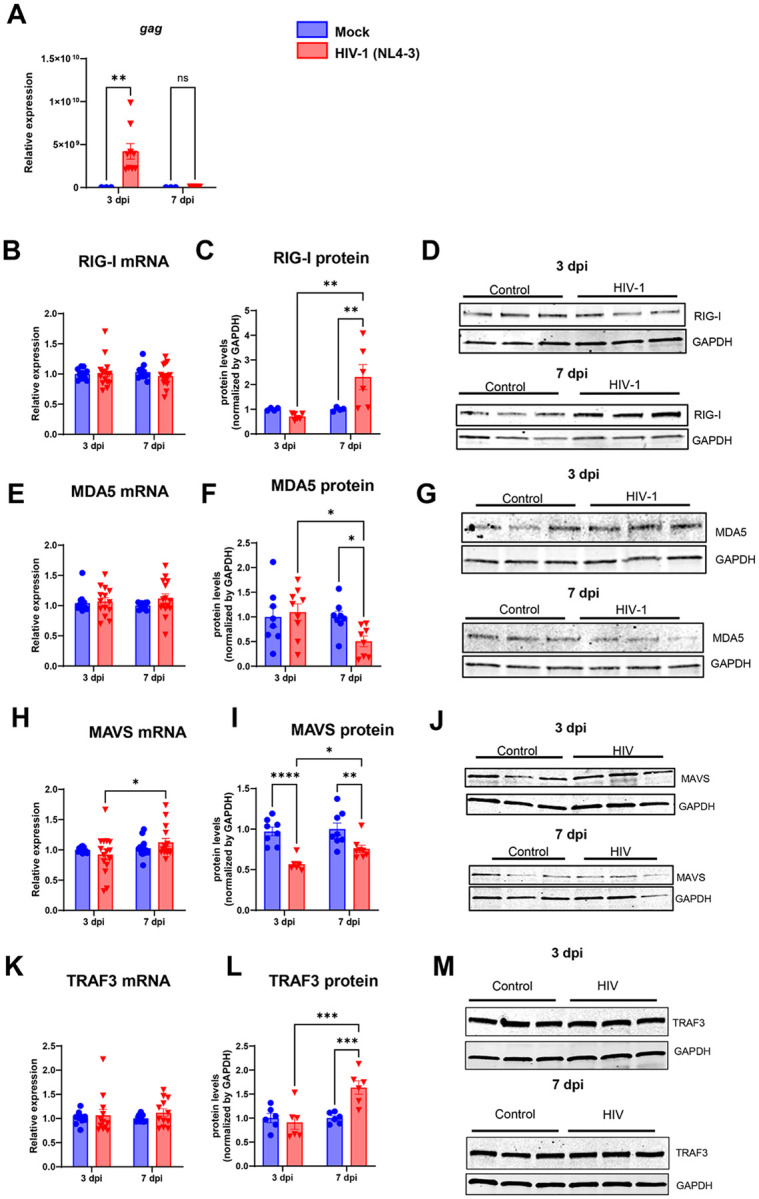
HIV-1 latent infection activates RNA sensors in human brain pericytes. Cells were infected with HIV-1 and mRNA was harvested at 3 and 7 days post-infection (dpi). At 3 dpi high levels of gag HIV-1 gene were detected in infected cultures, which was greatly reduced at 7 dpi. mRNA and protein levels of RIG-1 (**B-D**), MDA5 (**E-G**), MAVS (**H-J**) and TRAF3 (**K-M**) were determined by RT-qPCR and western blotting, respectively. *: 0<0.05; **: p<0.01, ***: p<0.001; ****: p<0.0001, Two-way ANOVA with Bonferroni post-test. Each dot in graphs correspond to an independent cell culture well.

First, human brain pericytes were infected with HIV-1 and analyzed at three or seven days post infection (dpi), which were shown by our group that correspond to active and latent infection times, respectively [26]. We confirmed HIV-1 replication rates by assessing *Gag* expression levels in human brain pericyte cultures by RT-qPCR. A significantly higher amount of Gag transcripts was found 3-days after HIV-1 infection when compared with Mock-infected cells. No increase in Gag levels were found after 7-days infection when compared with Mock infected pericytes (Figure 1A).

Because of the important role of the pattern recognition receptors (PRRs) and especially the RIG-I-like receptors (RLRs) in controlling viral RNA infections [40,41], we decided to study if the main genes involved in this pathway, RIG-1 and MDA5 could be differently affected during the active and the latent phase of HIV-1 infection. Our results showed that HIV-1 infection of human brain pericytes for 3- or 7-days did not alter the mRNA expression levels of these genes (Figure 1B and E). However, a significant increase in RIG-1 protein levels accompanied by a decrease in MDA5 protein levels was found at 7 dpi, at the time of latent infection when compared with 7-days Mock infected cells or with 3-days HIV-1 infected cells (Fig 1C–D, and F–G). No changes were found when pericytes were infected with HIV-1 for 3 days in RIG-1 or MDA5 proteins levels when compared with 3-days Mock infected cells (Figures 1C–D, and F–G).

RIG-1 signaling pathway includes the activation of the TNF receptor-associated factor (TRAF3) and the mitochondrial antiviral signaling protein (MAVS) proteins, generating a series of downstream events that include changes in the phosphorylation levels of IRF3, IRF7 and TBK1 [40,42–44]. We next questioned whether an HIV-1 infection in BBB pericytes could differentially regulate such downstream proteins in active versus latent infection. No changes were found in the mRNA expression levels of MAVS or TRAF3 genes after HIV-1 infection for 3 or 7 dpi when compared with uninfected control cultures (Figures 1H and K). However, we found a marked decrease in MAVS levels after HIV-1 infection at both 3 and 7 dpi, as compared to their respective uninfected controls (Figure 1 I–J). Conversely, TRAF3 protein levels were significantly increased at 7 dpi (Figure L–M).

**Figure 2:**
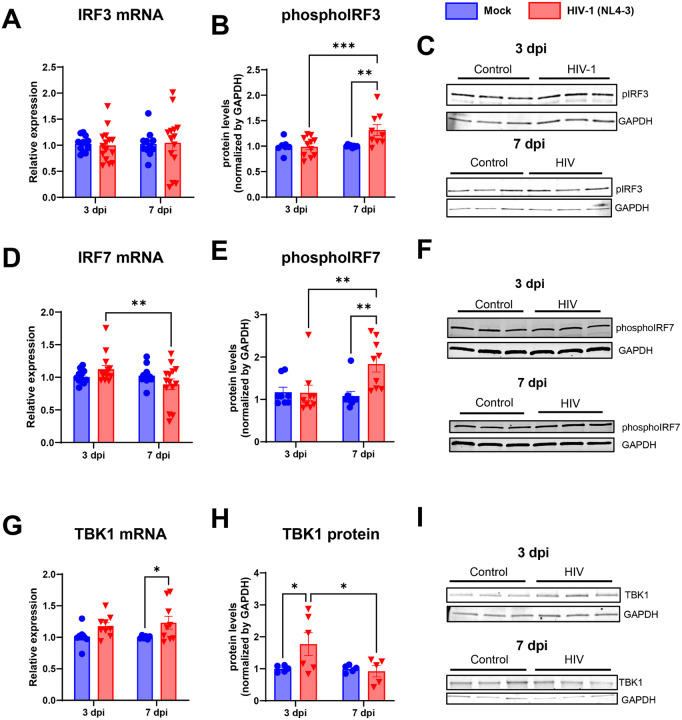
HIV transiently increases TBK1 protein but does not affect downstream IRF3 activation. TANK-binding kinase 1 (TBK1) is a signaling kinase that can be activated by RNA sensors and can lead to activate Interferon regulatory factor 3 (IRF3) transcription factor. TBK1 (**A** and **B**) and IRF3 (**C** and **D**) were analyzed by RT-qPCR (**A** and **C**) and western blotting (B and D). Representative blots for TBK1, phosphorylated IRF3 (pIRF3) and total IRF3 are shown in the right panels. *: p<0.05; **: p<0.01, Two-way ANOVA with Bonferroni post-test. Each dot in graphs corresponds to an independent cell culture well.

Consistent with TRAF3 upregulation, we found an increase in phosphorylated IRF3 (Figure 2B–C) and IRF7 (Figure 2E–F) at 7-days post HIV-1 infection, as compared to uninfected control cultures and with 3 dpi-infected cultures, although no significant changes were observed at the transcriptional levels for both IRF3 and IRF7 (Figure 2A and D). Furthermore, TBK1 mRNA levels were not affected at 3 dpi, but were significantly increased at 7 dpi by 1.23-fold (Figure 2G). At the protein level, HIV-1-infected cultures had a 1.77-fold increase in TBK1 protein contents, that were restored to control levels at 7 dpi (Figure 2H–I).

### 2. The cGAS-STING signaling pathway is temporally regulated in active and latent HIV-1 infection in human brain pericytes

Cyclic GMP–AMP synthase (cGAS)–stimulator of interferon genes (STING) signaling pathway is an essential mechanism of response to DNA by regulating inflammation after viral infections and cellular stress [45,46]. The cGAS–STING signaling pathway is involved in response to dsDNA but not canonically to RNA viral infections. We decided to study if this pathway could be also differently affected during the active and the latent phase of HIV-1 infection given that (a) it has been reported a strong functional interconnection between the RIG-1 signaling pathway and the cGAS–STING signaling pathway [47–49] and (b) the cGAS–STING signaling pathway is also involved in type I interferons (IFNs) expression and activation of IRF and TBK1 [50].

**Figure 3:**
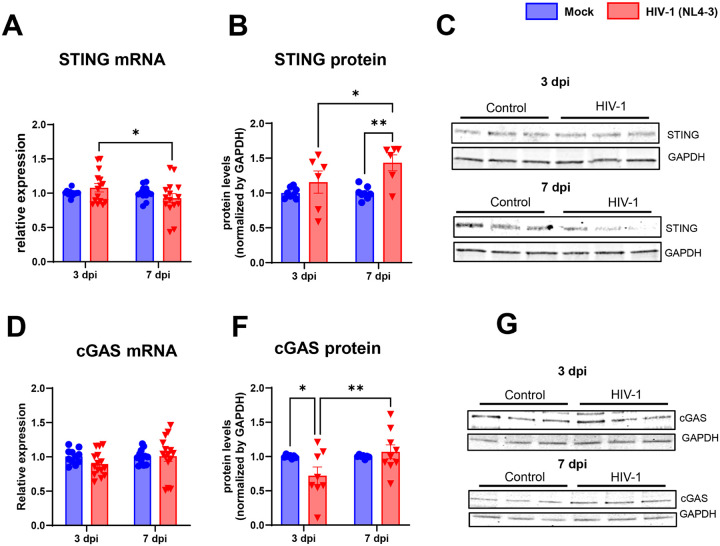
Latent HIV-1 infection activates STING-cGAS pathway in brain pericytes. Stimulator of IFN genes (STING) and cyclic GMP-AMP synthase (cGAS) mRNA (**A**and **C**) and protein (**B** and **D**) levels were analyzed 3 and 7 days post infection with HIV-1. STING (A-B) had a significant increase at 7 dpi, and cGAS had a reduction at 3 dpi (D), followed by an increase at 7 dpi (D). Representative blots for STING and cGAS are shown in the right panels. *: p<0.05; **: p<0.01, Two-way ANOVA with Bonferroni post-test. Each dot in graphs corresponds to an independent cell culture well.

In line with the results on RNA sensor pathways (RIG-1 and MDA5), STING and cGAS gene expression was not significantly altered in human brain pericytes infected with HIV-1 at 3 or 7 days as compared to uninfected cultures (Figure 3A and D). However, at 7 dpi STING was significantly increased at the protein levels as compared to uninfected controls (Figure 3B and C). Conversely, cGAS protein levels were significantly decreased at 3 dpi and restored to control levels at 7 dpi (Fig 3F and G).

### 3. Active and latent HIV-1 infection differentially regulates the expression of IFN genes and STAT signaling pathway in human brain pericytes

Activation of IRFs factors induces the expression of type I IFNs in a positive feedback. Once the IFNs cytokines are released, they bind to the interferon receptor complexes in the cell activating the Janus kinase signal transducer and activator of transcription (JAK/STAT) signaling pathway and starting the transcription of the IFN-stimulated genes (ISGs) [51,52]. We evaluated if HIV-1 infection could alter the JAK/STAT signaling pathway and the transcription of ISGs genes.

**Figure 4:**
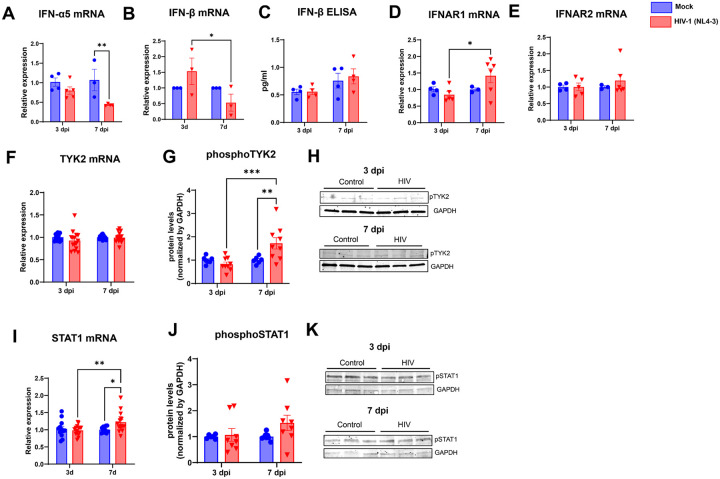
Legend not included with this version.

HIV-1-infected pericytes were analyzed at the active and latent phases (3 and 7 dpi, respectively) to assess the expression levels of several IFNs genes by RT-qPCR and ELISA. Our results indicated a significant decrease in IFNα5 mRNA levels 7 dpi in infected brain pericytes (Figure 4A) and a biphasic IFNβ mRNA expression response, with an increase at 3 dpi followed by a decrease at 7 dpi (Figure 4B). However, no significant changes were found at secreted IFN-β levels when measured by ELISA (Figure 4C).

**Figure 5:**
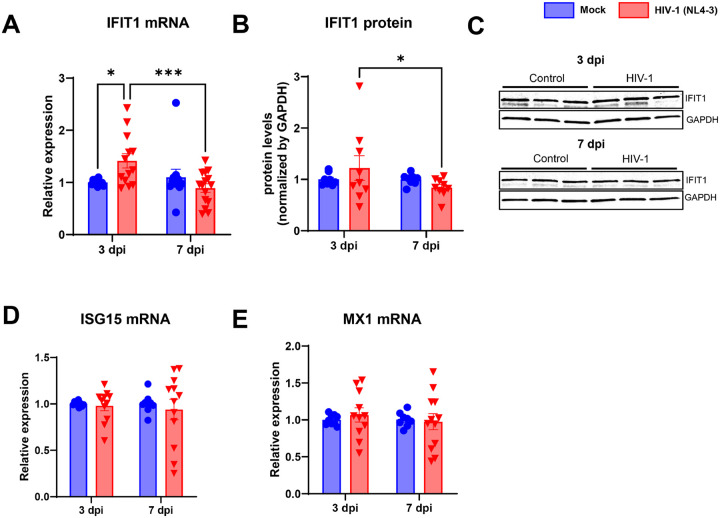
HIV-1 transiently increases innate antiviral response in pericytes. Expression of Interferon induced protein with tetratricopeptide repeats 1 (IFIT1) was assessed in human brain pericytes following HIV-1 infection. There was a transient increase in IFIT1 mRNA (**A**) and protein (**B**) expression at 3 dpi which was restored at 7 dpi. *: 0<0.05, ***: p<0.001, Two-way ANOVA with Bonferroni post-test. Each dot in graphs corresponds to an independent cell culture well.

IFN receptors IFNRA1 and IFNRA2 did not show any significant difference in expression following infection, although IFNAR1 levels were higher in infected dishes at 7 dpi as compared to infected ones at 3 dpi (Figure 4D–E). TYK2 is a member of the Janus kinase family and transduces signals from IFN receptors, which can lead to downstream activation of STAT signaling (reviewed by Morand et al., 2024). No changes to TYK2 mRNA levels were observed (Figure 4F), but phosphorylated TYK2 protein levels were significantly increased in 7 dpi-infected pericyte cultures (Figure 4G–H). STAT1 expression was also increased at 7 dpi cultures at the mRNA levels (Figure 5I), although this effect was not observed at the protein levels (Figure 4J–K).

Finally, we analyzed the expression of ISGs genes and showed a 1.41-fold increase in IFIT1 expression levels at mRNA 3 days after HIV-1 infection as compared with mock-infected cells (Figure 5A). IFIT1 protein levels were diminished in infected dishes at 7 dpi as compared to infected ones at 3 dpi (Figure 5B–C). No changes were found at mRNA expression levels of ISG15 and MX1 after HIV-1 infection (Figure 5D–E).

## DISCUSSION

Brain pericytes are a novel niche for HIV latency as a reservoir and given their role in regulating vascular tone and BBB physiology [53]. This work aimed at studying innate immune responses in these cells upon active and latent infection.

As part of a cellular innate response to RNA virus, the RNA helicases retinoic acid-inducible gene I (RIG-I) and the melanoma differentiation-associated gene 5 (MDA5) are the main cytosolic receptors that are responsible for the recognition of RNA [54]. We observed that while RIG-1 protein levels were increased in latently-infected pericytes, there was a significant decrease in MDA5. Interestingly, treatment with acitretin to induce RIG-1 activation led to increased apoptosis of HIV-1 latently infected CD4 + cells [55]. The mitochondrial antiviral protein MAVS signals downstream of RIG-I and serves as a platform for TBK1 activation. However, HIV-1 is capable of inhibiting MAVS expression via polo-like kinase 1 (PLK1) [56], which prevents the recruitment of TRAF3 and the downstream activation of type I IFN and cytokines responses [57, 58]. Accordingly, we observed that HIV-1 infected human brain pericytes had reduced MAVS protein levels, both at 3 and 7 dpi, which was consistent with the increased TRAF3 levels at 7 dpi.

We then evaluated the responses of two important RNA sensing proteins, the stimulator of interferon genes (STING) and cyclic GMP-AMP synthase (cGAS). The nucleotidyltransferase cGAS has emerged as a key sensor of cytoplasmic DNA species that are derived from various sources, including infection with DNA viruses as well as with retroviruses, which generate DNA through reverse transcription of their RNA genomes. Since its discovery, cGAS has been thought to recognize cytoplasmic mislocalized double-stranded DNA (dsDNA) in a sequence-independent manner [59], but its relevance in HIV-1 natural human infection is still uncertain and variable according to cell type [60]. Activated cGAS catalyzes the cyclization of ATP and GTP, resulting in the small molecule and cyclic dinucleotide cGAMP [45, 61]. cGAMP induces a STING/TBK1/IRF3-dependent signaling cascade that culminates in the expression of interferon-regulatory factor (IRF)–induced genes and type I IFNs. HIV-1-infected brain pericytes had increased STING protein levels at 7 dpi and a transient cGAS decrease at 3 dpi, reinforcing the differential responses to active and latent HIV infection to RNA sensing and IFN responses in

After viral infection, the immune system starts the production of antiviral cytokines, being IFN [10] the most powerful one. There are three families of IFN, Types I, II and III, being IFNα/β the best-defined and most broadly expressed type I IFNs. These cytokines are known to inducing an antiviral state, triggering a gene expression program that interferes with viral replication stages through various mechanisms [62]. In humans, type I IFN (IFN-α) response was shown to correlate with progressive HIV and CD4 + T cell death [63], and plasma levels of TNF-α and Tumor necrosis factor (TNF)–related apoptosis-inducing ligand (TRAIL), a TNF superfamily member, were increased in HIV-1 patients [64].

IFNα/β production occurs in response to the stimulation of receptors that recognize pathogen-associated molecular patterns (PAMPs), including STING and the RIG-I-like family [65]. Although IFN-α5 and IFN-β transcripts were significantly reduced in latently infected pericytes, no changes at IFN-β secretion were observed herein.

IFN α/β production has bystander effects on neighboring cells, that express IFNRA1 and IFNAR2 receptors. Upon activation, IFNARs induce activation of receptor-associated protein tyrosine kinases Janus kinase 1 (JAK1) and tyrosine kinase 2 (TYK2). JAK1 and TYR2 activation leads to phosphorylation of signal transducer and activator of transcription 1 (STAT1) and STAT2 molecules that are present in the cytosol, leading to the dimerization, nuclear translocation and binding of these molecules to IRF9 to form the ISG factor 3 (ISGF3) complex. Our data showed that IFNRA1 mRNA expression was increased in latently infected cultures as compared to actively infected ones. Interestingly, chronically infected myeloid U1 and OM10.1 cell lines have reduced IFNAR1 expression, indicating that brain pericytes may respond differentially to HIV latent infection [66]. Accordingly, phosphorylated TYK2 levels were increased in latently infected pericytes, suggesting increased IFN response signaling in latently infected cells.

Finally, as part of the group of IFN stimulated genes (ISGs), we evaluated expression of a member of the interferon-induced proteins with tetratricopeptide repeats family IFIT1. In human macrophages, HIV-1 induces a sustained increase in IFIT1 expression from 3 to 9 days post infection [67]. Herein, IFIT1 was transiently increased in actively infected brain pericytes, being reduced at 7 dpi, while no changes to ISG15 and MX1 transcripts were described in infected cells.

We conclude that latently infected pericyte cultures sustained higher levels of RNA sensor proteins as well as IFN regulated genes. These findings may represent novel avenues for modulating reservoirs in the CNS and prevent HAND manifestations.

## Figures and Tables

**Table 1.** Antibodies used in this study

Name	Company	Code	Host	Molecular Weight	Dilution
cGAS Monoclonal	Thermo	14-5158-82	Mouse	65 KDa	1:250 (worked)
IFIT1	ABCAM	ab70023	Mouse	56 KDa	1:500–1:1000
IRF-3	Cell Signaling Technology	4302S	Rabbit	45–55 KDa	1:1000
IRF-7	Cell Signaling Technology	4920S	Rabbit	65 KDa	1:1000
MAVS	Proteintech	14341–1-AP	Rabbit	57 KDa	1:2000–1:6000
MDA5	ABCAM	ab283311	Rabbit	117,140 KDa	1:1000
phosphoIRF-3 (S396)	Cell Signaling Technology	4947S	Rabbit	45–55 KDa	1:1000
phosphoIRF-7 (S471/472)	Cell Signaling Technology	5184S	Rabbit	65 Kda	1:1000
Phospho-STAT1 (Tyr701)	Thermo	700349	Rabbit	100 KDa	1:250–1:1000
Phospho-STAT1B (Ser727)	Thermo	44–382G	Rabbit	100 Kda	1:1000
phosphoTBK1/NAK (S172)	Cell Signaling Technology	5483S	Rabbit	84 Kda	1:1000
RIG-I	Thermo	700366	Rabbit	150 KDa	1:500
STAT1	Thermo	MA1–037	Mouse	100 KDa	1:1000
STAT1	Thermo	PA5–81911	Rabbit	100 KDa	1:500–1:1000
STING	ABCAM	ab239074	Rabbit	37–42 KDa	1:1000
TBK1/NAK	Cell Signaling Technology	51872S	Mouse	84 Kda	1:1000
TRAF3	ABCAM	ab239357	Rabbit	64 Kda	1:1000
RIG-I Recombinant Rabbit Monoclonal Antibody	Thermo	700366	Rabbit	150 Kda	1:500
STAT1 Monoclonal Antibody (15H3)	Thermo	MA1–037	Mouse	100 Kda	1:1000
STAT1 Polyclonal Antibody	Thermo	PA5–81911	Rabbit	100 Kda	1:500–1:1000
STING [EPR13130–55]	ABCAM	ab239074	Rabbit	37–42 Kda	1:1000
TBK1/NAK (E9H5S) Mouse mAb	Cell Signaling Technology	51872S	Mouse	84 Kda	1:1000
TRAF3	ABCAM	ab239357	Rabbit	64 Kda	1:1000
